# Season Suspension and Summer Extension: Unique Opportunity for Professional Team-Sport Athletes and Support Staff During and Following the COVID-19 Crisis

**DOI:** 10.3389/fspor.2020.00098

**Published:** 2020-07-07

**Authors:** Thomas Huyghe, Stephen Bird, Julio Calleja-González, Pedro E. Alcaraz

**Affiliations:** ^1^Research Center for High Performance Sport, Centro de Investigación en Alto Rendimiento Deportivo, Universidad Católica de Murcia, Murcia, Spain; ^2^School of Health and Wellbeing, University of Southern Queensland, Toowoomba, QLD, Australia; ^3^Department of Physical Activity and Sports, Faculty of Education, Sports Section, University of the Basque Country, Vitoria-Gasteiz, Spain

**Keywords:** COVID-19, team sports, physical preparation, periodization, virtual training, athlete monitoring, pandemic

## Abstract

Given the emergence of the COVID-19 outbreak, the official declaration of a global pandemic by the World Health Organization, and its consequential disruptions on the professional team sports landscape, it is the main objective of this brief opinion to help ensure that professional team-sport athletes and support staff remain aware as a society of some of the potential pitfalls – illustrated via negative but plausible detrimental scenarios. Finally, new ideas are introduced and evidence-based hypotheses are presented on the following five themes: periodization, exploration, virtual (at-home) training, player monitoring, and continued education, including return-to-competition preparation strategies following these exceptional times of uncharted territory.

## Season Suspension

In 2020 the World witnessed the emergence of a novel, viral, zoonotic pathogen (SARS-CoV2) leading into an outbreak of the Coronavirus disease 2019 (COVID-19) (Rodríguez-Morales et al., 2020a) which forced the World Health Organization to declare a Public Health Emergency of International Concern (PHEIC) (Biscayart et al., 2020; Rodríguez-Morales et al., 2020b) and profile this disease as a global pandemic (Wan, 2020). As of June 23, 2020, 9,218,565 cases were confirmed worldwide causing 474,966 deaths (Covid-19 Coronavirus Pandemic, 2020). As time goes by, global concern and cumulated cases have increased (Covid-19 Coronavirus Pandemic, 2020; Hui et al., 2020; Wang et al., 2020). Consequently, the International Olympic Committee and Japan's Prime Minister, Shinzo Abe announced that the Tokyo 2020 Olympic games will be postponed until 2021 (Rich et al., 2020). Since the opening of the first modern Olympic Games in 1896, the international sports competition has only been canceled 3 times: once during World War I (1916) and twice during World War II (1940, 1944) (Rich et al., 2020; Roos, 2020), which reflects the unprecedent nature of our current COVID-19 crisis.

Furthermore, this rare and unknown situation has evoked drastic changes and disruptions in the professional team sports landscape around the world, including the suspension or even cancellation of the 2019–2020 season in an attempt to curb the spread of the fatal virus. For instance, repercussions were publicly reported on some of the world's most popular professional team sports leagues. In particular, the National Basketball Association (NBA) suspended the season on March 11, 2020 after NBA player Rudy Gobert tested positive for COVID-19 and is using a 30-day hiatus to determine next steps (Aschburner, 2020). This pivotal decision by the NBA's Commissioner, Adam Silver, has instantly set off a chain of events that led to the postponement of several high-profile team sport events for millions of people (Zillgitt, 2020), including the NCAA men's and women's tournaments, the NHL season, MLB's spring training, the English Premier League season, La Liga season, Bundesliga season, Serie A season, UEFA's European Championship, and Ligue 1 season, to name a few.

Given the worldwide suspensions of the ongoing 2019–2020 regular season, professional team-sport organizations are facing unprecedented times which require diligent analysis, evaluation, and adaptations to cope with the altered schedule, potential deconditioning of their athletes, and high level of uncertainty (Mujika and Padilla, 2000,?; Jaspers et al., 2018; Corsini et al., 2020). However, to the best of authors' knowledge, scarce information is known on how to optimally cope and prepare professional team-sport athletes and support staff during these unfamiliar times and isolated conditions (Nieman and Wentz, 2019; Corsini et al., 2020). Therefore, new ideas are briefly introduced in the following sections with the intent to help professional team-sport athletes and support staff in preparing their upcoming endeavors to the best of their current capabilities.

## Summer Extension

Considering the current state of the COVID-19 outbreak, it is increasingly probable that massive international events, including the examples mentioned above, will be further postponed or canceled entirely until we begin to see regression and resolution of this outbreak in due course (Ahmed and Memish, 2020). While moving forward through this uncertain period of time, professional team-sport athletes and support staff may aim to anticipate various plausible scenarios, such as: team practice time constraints upon return to competition, limited accessibility to fitness and rehabilitation equipment, prolonged social and physical distancing, financial deficits, limited personal space, to name a few. Regardless of how the current situation unfolds, each scenario will require proactive and personalized interventions with respect to both physical and mental aspects of team-sport athletes' preparedness.

Although our ability to extrapolate research findings from similar historic periods of “summer extension” on the health and performance of professional team-sport athletes is questionable given the exceptionality of the current pandemic, it is well-known that the restriction to sport-specific stimuli due to the disruption or suspension of the competition calendar can evoke serious complications (Myer et al., 2011; Sarto et al., 2020). For instance, the National football League players had limited access to facilities and training resources because of a competition lockout from 11 March 2011 to 25 July 2011, which conjured a significantly higher rate of Achilles tendon injuries accrued over the first period of the training camp and the subsequent season (Myer et al., 2011; Sarto et al., 2020). In light of this concern and potential harm to athletes, a Google Forms survey was distributed to African elite and semi-elite athletes (692 respondents) from 15 sports (last week of April 2020) at level 5 of the lockdown period (Pillay et al., 2020). In this report, it appeared that most athletes trained alone (61%; *p* < 0.0001), daily (61%; *p* < 0.0001) at moderate intensity (58%; *p* < 0.0001) and for 30–60 min (72%), preferred sedentary behavior over active behavior in their leisure time (*p* < 0.0001), sleep patterns changed significantly (79%; *p* < 0.0001), and most athletes consumed disproportionate amounts of carbohydrates (76%; *p* < 0.0001), felt depressed (52%), and required strong encouragement to hold an active lifestyle (55%) (Pillay et al., 2020). Recognizing the abovementioned concerns and initial report on COVID-19, researchers have reiterated the importance and urgency for increased awareness to how professional team-sport organizations can prepare for “the new normal,” including adjustments in sports programming (e.g., need for a prolonged preseason) (Sarto et al., 2020) and extensive cardiac screening protocols (e.g., echocardiography, stress testing, rhythm monitoring) (Phelan et al., 2020; Schellhorn et al., 2020).

**Figure 1:**
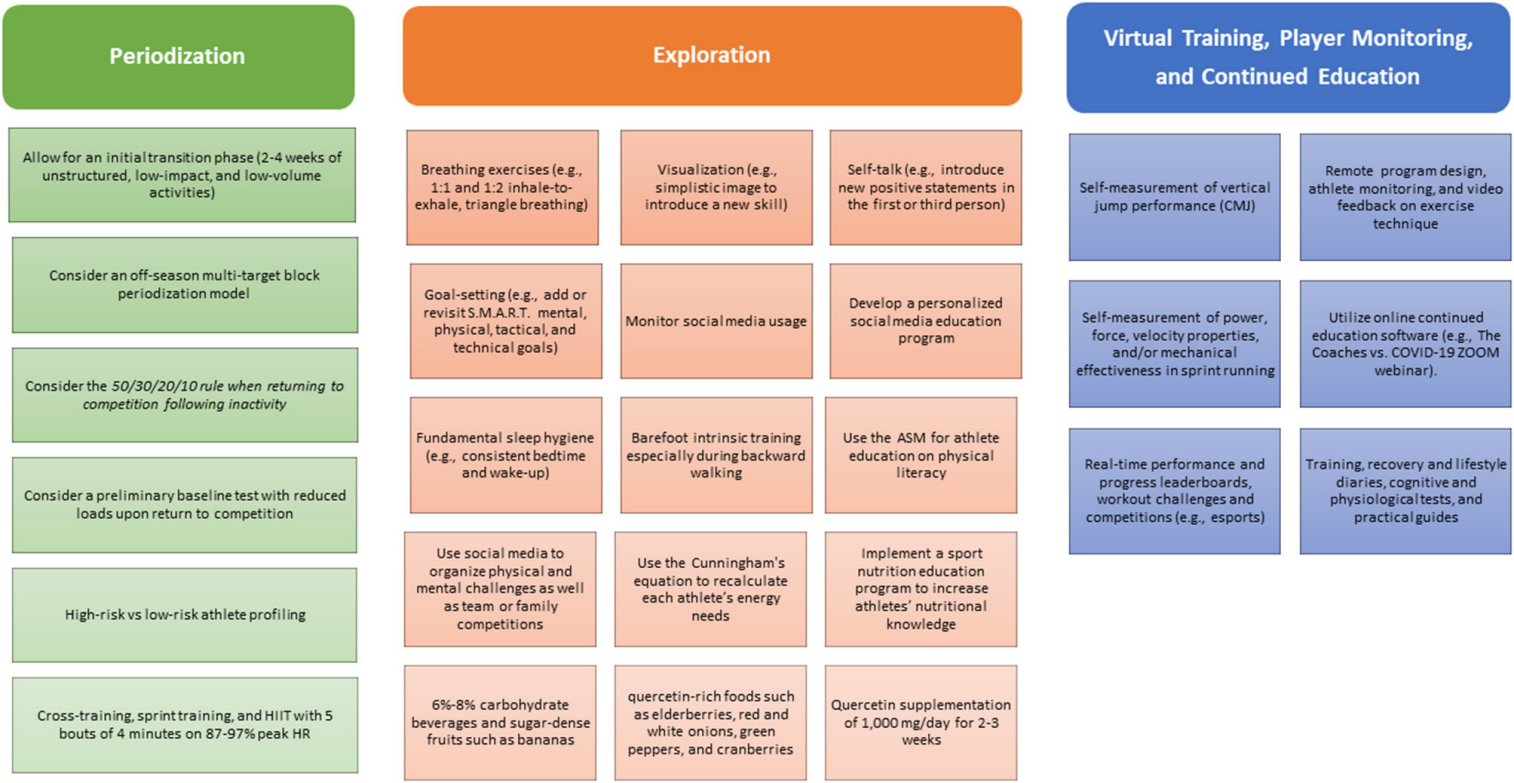
Three unique opportunities for team-sport athletes during the COVID-19 Crisis.

With diligent respect to the remaining uncertainty of the COVID-19 pandemic, this article provides suggestions and critical reflections for professional team-sport athletes and support staff in order for them to reframe these challenges as “an opportunity to evolve,” with particular attention to five performance themes: periodization, exploration, virtual training, player monitoring, and continued education (Figure 1).

## Unique Opportunities for Team-Sport Players

### Periodization

Although uncertainty remains on whether, and which, current professional team-sport competitions will completely terminate or resume, unique opportunities inherently reside in the midst of these challenging times. For instance, limited exposure to organized sport-specific practices may allow strength and conditioning coaches to design a robust long-term periodization plan tailored to the personal needs of each athlete. Presupposing that league executives take into account the necessary (re)training time (“minimum effective dose”) required for athletes to regain optimal physical conditions and maintain or at least attenuate the decay of endurance- and neuromuscular-related performance parameters upon return to training and competition (Silva et al., 2016), strength and conditioning coaches may consider a multi-target block periodization model (i.e., the development of many targeted abilities within sequenced block mesocycles containing a minimal number of compatible training modalities) (Issurin, 2016). Given that sports require the expression of multiple athletic abilities at the same time (28 studies), multi-targeted block periodization has revealed their distinct superiority compared with traditional preparation strategies (i.e., the administration of highly concentrated training means for enhancement of one leading fitness component) (Issurin, 2016). However, prior to structured planning of (re)training, an initial transition phase (2–4 weeks of unstructured, low-impact, and low-volume activities) (Issurin, 2016; Caterisano et al., 2019) could be prescribed to allow certain athletes (e.g., high-minute players) to recover, mentally relax, regenerate and alleviate chronic microtrauma accumulated over the season due to repetitive single-sport participation (Mujika and Padilla, 2000; Lemont et al., 2003; Irving et al., 2008; Cook and Purdam, 2009; Monto, 2012). As many team-sport athletes may have had limited chances to fully recover from extensive competition periods in the past (e.g., national team games, playoffs, prolonged preseason, etc.), the potential benefits of incorporating a transition period during the initial stage of the current suspension period (e.g., happiness) (Calleja-González et al., 2018) may outweigh the potential risks involved (e.g., short-term detraining), especially considering the likelihood of continued postponement of competition until further notice. Ultimately, the transition phase should prepare and rejuvenate athletes for the subsequent training regimens (i.e., accumulation phase consisting of 2–4 weeks of progressive overload training focused on basic fitness qualities such as aerobic intermittent endurance, muscle strength adaptation, and general coordination) (Issurin, 2016). In turn, this enables professional team-sport athletes to (re)acquire fundamental workload tolerability and adaptation. Hereafter, strength and conditioning coaches may carefully examine and design the construct of relatively higher loading phases (i.e., transmutation transformation and realization phase) (Issurin, 2016).

Although gradual training overload including high-intensity exercise bouts are necessary and self-evident as part of the return-to-competition process, as long as COVID-19 vaccines remains to be revealed and unfolded, careful attention should be given to the introduction of “accumulation training phases” given that prolonged periods of high-volume and high-intensity training may adversely increase the risk of illness among athletes (Nieman and Wentz, 2019; Corsini et al., 2020). Even when the WHO may announce a gradual regression of their containment policies, as supported by a sustained reduction in the number of new infections, hospitalizations, and having sufficient nation-wide supply of COVID-19 testing kits, strength and conditioning coaches should still remain alert for potential risk of injury (Corsini et al., 2020) for the following reasons: almost 60% of non-contact injuries have been reported during periods in which collegiate athletes transitioned back into training following a period of inactivity (e.g., after vacation) (Caterisano et al., 2019); cardiac atrophy may occur as soon as 4 weeks of detraining (Pelliccia et al., 2002; Pedlar et al., 2017); an overall loss of 10% of fitness for each week of total inactivity can be generally expected (Eirale et al., 2020); loss of lean mass and muscle strength represent an important injury risk factor (Casa et al., 2012); and the risk for serious injury, and death after a period of inactivity is well-documented (Casa et al., 2012; Caterisano et al., 2019). Recognizing the potential risks involved with reduced training, incorporating cross-training (Mujika and Padilla, 2000,?), sprint training (Joo, 2018), and high-intensity interval (HIIT) training sessions (5 bouts of 4 min on 87–97% peak heart rate) every second week of the “at-home period” may aid in the preservation of the athlete's threshold to transport and use oxygen during physical activity (VO2^max^) and repeated sprint ability (RSA), as previously demonstrated in soccer athletes (Slettaløkken and Rønnestad, 2014; Joo, 2018), and in turn, minimize the potential drop-off in cardiorespiratory fitness as a potential consequence of the COVID-19 pandemic.

In light of this concern, the “50/30/20/10 rule” spanning over a 2–4 week training period may serve as a useful baseline approach to individual and team load management, as reported by a joint consensus between the National Strength and Conditioning Association (NSCA) and Collegiate Strength and Conditioning Coaches Association (CSCCa) in 2019 (Caterisano et al., 2019). In particular, with new athletes entering the program, it is advised to reduce the overall conditioning volume by at least 50% of the uppermost conditioning volume on file in the first week following inactivity, and by 30, 20, and 10% in the following 3 weeks, respectively, with a 1:4 or greater work: rest ratio in the first week, and a 1:3 work: rest ratio or greater in the second week (Casa et al., 2012). Furthermore, the “FIT rule” (frequency, intensity volume, and time of rest interval) may offer a useful guideline for resistance training programs following periods of inactivity (Casa et al., 2012). Furthermore, these evidence-based recommendations align with previously published statements by the National Athletic Trainers' Association (NATA), the National Collegiate Athletic Association (NCAA) Committee on Competitive Safeguards and Medical Aspects of Sports, the NCAA Division I Football Oversight Committee, the United Kingdom Strength and Conditioning Association (UKSCA), Australian Strength and Conditioning Association (ASCA), and several other scientific, medical, and sports organizations, and are grounded on the emerging scientific agreement (Caterisano et al., 2019).

Importantly, strength and conditioning coaches should tailor the abovementioned prescriptions toward their environmental conditions and the individual needs of each athlete to ensure adequate ecological validity of their interventions (Caterisano et al., 2019). For instance, careful attention should be given to high-risk profile (more fragile) athletes (i.e., athletes with a lower threshold of fatigability, burnout, or muscle failure) to protect their health and achieve the desired adaptations (Windt et al., 2017). In particular, athletes who maintain their baseline aerobic fitness level and lack of injury history, may tolerate higher training workloads given that their associated risk for injury is lower compared to the average athlete (Windt et al., 2017). In contrast, the allowable personal workload “threshold” for more fragile athletes may be lowered with less aggressive overloading procedures to evoke safe and optimal adaptations (Windt et al., 2017). Therefore, it is extremely important for professional team-sport support staff to measure the baseline impact of the confinement period on each athlete's state of fitness and overall well-being upon return to organized group activities (Eirale et al., 2020).

Organizationally, once governing bodies offer “green light” to professional team sport organizations to return their activities, it is highly recommended to apply a progressively programmed approach (Eirale et al., 2020). In this regard, a 4-phase protocol has been suggested most recently, which consists of: remote training program at home designed and monitored by the team's performance support staff (phase 1), individual in-person training program in which the athlete is assisted by a few team's performance support staff members ensuring safe physical distance (phase 2), in-person group training program in which maximum 8 athletes are trained at once (phase 3), in-person group training program for the entire team (phase 4) (Eirale et al., 2020).

Finally, during as well as following the return-to-play period, rigorous medical and hygienic-sanitary rules should be clarified, emphasized, and enforced for all members of the organization prior to recommencement. In this sense, the following regulations should be addressed and will remain critical in our battle against potential COVID-19 transmissions: washing hands frequently with soap and water for at least 20 s using hand sanitizer with at least 60% alcohol, practicing physical distances of ~6 feet or more whenever possible, minimize domestic and international air travel (especially public flights), ensure entry screening procedures (e.g., airport, gyms, practice facilities) of body temperature and potential symptoms of COVID-19 (i.e., fever, pneuomonia, fatigue, dry cough, myalgias, loss of smell, dysgeusia, sore throat, rhinorrhea), and isolating athletes with confirmed or suspected COVID-19 symptoms (and those who came in close contact with them in the preceding 14 days) (Toresdahl and Asif, 2020).

Even though the abovementioned strategies may likely evoke positive results, the novelty of the current situation in which professional team-sport organizations are residing, warrants future investigations in order to confirm the effectiveness of these implications so that, if and when, similar events may (re)occur in the future, professional team-sport athletes and support staff are strategically equipped and fully prepared (i.e., emergency action plan).

### Exploration

Team-sport athletes and support staff may explore new ways in developing their craft by the incorporation of training and recovery interventions that might have been undermined during the course of the season due to the congested nature of the competition calendar (e.g., travel, games, few practices, and media requirements) (Esteves et al., 2020; Huyghe et al., 2018). For instance, the following remote training and individual recovery modalities may serve as an opportunity for supplemental thought and consideration during the COVID-19 crisis:

- Based on the first immunological model of COVID-19 supported by an in-depth scientific report published on May 2nd of 2020 in the Journal of Pediatric Allergy and Immunology (Matricardi et al., 2020), the risk and severity of COVID-19 symptoms and potential fatality in healthy populations derives from the balance between two core parameters: (1) their overall level of viral exposure and (2) the efficacy of the local innate immune response (i.e., natural IgA and IgM antibodies, mannose-binding lectin) (Matricardi et al., 2020). This scientific framework is of critical importance for the professional team-sport community for the following two reasons: (1) the levels of salivary IgA tends to decline in athletes during and after a training session, leaving an open window for infection between 3 and 72 h after strenuous activities (Gleeson et al., 1999); (2) extreme physical activity and oral breathing with hyperventilation during the incubation days and early stages of COVID-19 promotes the re-inhalation and early direct penetration of high numbers of COVID-19 particles (from personal and teammates' or opponents' aerosol) in the lower airways and the alveoli which enables the virus to bypass the efficient immune barrier of the upper airway mucosa in already infected athletes (Matricardi et al., 2020). Hence, this clearly explains why professional team-sport athletes may be at an inherently higher risk of upper airway infections compared to semi-professional athletes and/or the moderate-active populations. Given that the amount of virus particles reaching the lungs is a determining factor deciding the fate of the athlete (Matricardi et al., 2020), and considering that oral pharyngeal breathing is the predominant mode of breathing during strenuous exercise (Pierson, 1989) while ventilatory rate increases (Pierson, 1989), professional team-sport coaches and support-staff may encourage and (r)emphasize the (re)training of optimal breathing techniques to reduce the amount of viral particles that can penetrate the lungs of their athletes, and in turn, reduce the chance of potentially severe symptoms or complications (e.g., pneumonia). In this regard, the combination of cold exposure training and a hyperventilation breathing exercises (while adhering the required physical distance distance) might be a reasonable strategy to consider given it can help support non-infected athletes' natural defense mechanisms (i.e., promotes attenuation of endotoxin-induced inflammatory responses) (Kox et al., 2020).
- Besides the implementation of breathing exercises to strengthen the immune system, slow diaphragmatic breathing can be considered as a practical and easy-to-teach tool to give athletes an edge on successfully managing stress reactivity (mentally and physically), whether they are preparing for a game or important event, or still recovering from strenuous activities (Martarelli et al., 2011; Hunt et al., 2018).
- Considering that respiratory pathologies such as asthma, chronic cough, recurrent respiratory infections and various upper airways conditions are common in elite athletes, and often underdiagnosed and undertreated (Boulet, 2012), and that air pollution can have a detrimental effect on athletic performance (Pierson, 1989), early awareness and careful examination of air pollutants as well as the abovementioned “subtle” respiratory pathologies should remain an integral and consistent part of the screening procedures employed by professional team-sport organizations. Additionally, these conditions should be recognized and treated according to evidence-based guidelines including the restrictions and prescriptions of drugs for therapeutic use of these conditions (Boulet, 2012).
- Given anxiety in elite athletes broadly reflect those experienced by the general population (e.g., higher anxiety in younger athletes, injured athletes, athletes who experienced 1 or more recent adverse life events) (Rice et al., 2019) and the COVID-19 crisis has evoked anxiety, rumination, frustrations, and loneliness in numerous athletes (Schinke et al., 2020), personal development (Jukic et al., 2020), monitoring mental state (Jukic et al., 2020), and mental skill development (Esteves et al., 2020) could be (re)trained and (r)emphasized. Particularly, special attention may be given to breathing (e.g., 1:1 and 1:2 inhale-to-exhale, triangle breathing), visualization (imagery) (e.g., simplistic image to introduce a new skill), self-talk (e.g., introduce new positive statements in the first or third person), and goal-setting (e.g., add or revisit S.M.A.R.T. mental, physical, tactical, and technical goals) strategies (Hunt et al., 2010). This guided approach is especially important as a lack of directions during the COVID-19 may result in athletes suffering from psychological stress, burnout, personal feelings of alienation, and mental illness (Schinke et al., 2020). Therefore, accessibility to telehealth communication and strong lines of support with sports psychologists should be regarded as a critical opportunity for long-term investment for professional team sport organizations (Jukic et al., 2020; Schinke et al., 2020).
- Identify underlying elements associated with mental distractions imposed by social media usage, including positive and unwanted messages, branding pressures, and competitor content, and subsequently, develop a personalized social media education program (Hayes et al., 2020). For instance, interventions may include: switching off and handing over the control of social media accounts, engage athletes through self-awareness and challenging their distractions, use social media to monitor the mood and psychological state of athletes across the current crisis (Hayes et al., 2020).
- Fundamental sleep hygiene strategies could be (re)introduced such as the (r)enforcement of consistent bedtime and wake-up times to ensure the athlete's circadian clock remains aligned with his/her natural environment during this crisis, and in turn, help him/her with building optimal long-term sleep habits that may transcend back to the in-season competition period (Bird, 2013; Lalor et al., 2020).
- Given neuromuscular and proprioceptive training likely moderates the risk of ankle and knee injuries (Dargo et al., 2017) and likely improves sports performance (Han et al., 2015), barefoot intrinsic training may be introduced into the athlete's training program as it allows greater sensory information feedback from the plantar area resulting in better biomechanical behavior, especially during backward walking (Sun et al., 2020).
- Given the positive impact of “physical literacy” (i.e., teaching fundamental motor skills) on lowering the risk of sports-related injuries, improving sports performance, and serving as a foundation for expertise and improve the overall quality of life in both athletes and non-athletes (Roetert et al., 2018; Savelsbergh and Wormhoudt, 2018), this concept can be (r)emphasized during our current crisis to enrich the quality of our own lives as well as those around us (Roetert et al., 2018). For instance, athletes may use social media platforms to demonstrate their new skills to the world and compete with others (e.g., their teammates) (Zwolski et al., 2017). More specifically, practitioners may consider the Athletic Skill Model (ASM) as a suitable framework for athlete development through movement education and creating personally challenging environments (Savelsbergh and Wormhoudt, 2018). Ultimately, this may benefit athletes not only during the course of their career, but also thereafter by means of lifelong physical activity.
- Given team-sport athletes may experience a lowered resting basic metabolic rate (BMR) as a consequence of a lowered daily energy expenditure due to various uncontrollable factors (e.g., absence of team practices, lack of out-home travel) (Kim et al., 2009), the Cunningham's equation may be an appropriate method for recalculating each athlete's energy needs (Kim et al., 2009), and in turn, provide personalized instructions for food and fluid consumption during these unusual times to ensure an adequate body composition. In this sense, athletes may spend more time learning how to cook as they may be restricted in chefs and teams providing their typical daily meals. Subsequently, professional team-sport support staff could implement a sport nutrition education program to increase the nutritional knowledge and nutritional status of their athletes (Rossi et al., 2017) as well as reduce their body fat percentage, total fat mass, and increase lean muscle mass, strength, and jump ability (Holway and Spriet, 2011). Furthermore, assessment of maturity status and simple anthropometric measurements (e.g., body mass scale, urine specific gravity refractometer) can help identify players prone to dehydration (Holway and Spriet, 2011), and in turn, provide personalized solutions to regain their optimal hydration status.
- Scientifically grounded nutritional and pharmacological strategies to prevent, relieve and/or combat the COVID-19 virus, as well as to accelerate the recovery following the potential contraction of COVID-19 virus, remains unclear. However, the following nutritional and pharmacological strategies may aid in fighting inflammation, preventing common infectious diseases, and alleviating states of immunodeficiency among professional team-sport athletes and support staff.
  ◦ Carbohydrate (CHO) intake before and during extensive and intensive exercises (e.g., 6–8% CHO beverages, sugar-dense fruits such as bananas) is associated with reduced level of stress hormones, blood levels of neutrophils and monocytes, and reduces inflammation (Nieman, 2010);
  ◦ Polyphenols (e.g., quercetin-rich foods such as elderberries, red and white onions, green peppers, and cranberries) combined with green tea extract, isoquercetin, and fish oil play a significant role in the reduction of inflammation following exercise with chronic support of innate immune function in athletes (Nieman, 2010);
  ◦ Quercetin supplementation (1,000 mg/day for 2–3 weeks) reduces illness rates in exercise-stressed athletes (Nieman, 2010);
  ◦ Given the COVID-19 pandemic may elicit overall reductions in physical activity and thus energy expenditure, an increased intake of daily protein (i.e., 2.3 g/kg body mass) may mitigate lean muscle mass loss during periods of reduced caloric intake (Jukic et al., 2020) with an emphasis on leucine as a fundamental amino acid in muscle protein synthesis (Jukic et al., 2020).
  ◦ In case of absence of fever, intake of NSAIDs (e.g., paracetamol, ibuprofen) and corticosteroids should be avoided given the potential negative effects they may elicit in the onset or masking of viral infections (Eirale et al., 2020; Toresdahl and Asif, 2020). Although more robust research is needed on this topic, a cautionary approach in their use is recommended (Eirale et al., 2020; Toresdahl and Asif, 2020).
- Given that infections can disrupt team-sport training and performance, and understanding that the risk for infections among team-sport athletes and support staff stem from a compounding effect of stressors (Kim et al., 2009), it would be appropriate and reasonable for professional team-sport organizations to consider the implementation of a personal “immune system scorecard” (e.g., visualization of risk status through a color-coded traffic light system) during and following the COVID-19 crisis. This scoring card may present key outcome measurements of overall immune system health such as secretory immunoglobulin A (SIgA) (Kim et al., 2009), prominent indicators of COVID-19 emergency (e.g., core body temperature) (Toresdahl and Asif, 2020), main risk factors of COVID-19 lethality (e.g., age) (Toresdahl and Asif, 2020), alongside a “magnitude score” on the exposure to common potential “illness stressors” involved with professional team-sport participation, such as: travel (duration, frequency, altitude, time zones crossed), psychological stress (daily wellness questionnaires, level of anxiety), sleep quality and quantity (e.g., sleep questionnaires and diaries), team compliance (e.g., checklist for cleaning duties, physical contact, sharing of personal belongings, oral, dental, feet, and hand hygiene, manifestation of COVID-19 symptoms, household sickness), nutrition and hydration state (e.g., calorie intake, energy balance, supplement use, alcohol use), environmental conditions (season, humidity, air quality, room temperature, outside temperature), and relative workload exposure (e.g. monotony, strain) (Nieman, 2010; Keaney et al., 2018). Nonetheless, future research is required (e.g., mass spectrometry) to better elucidate how stressors individually and collectively influence immunity against the COVID-19 virus as well as other illnesses in professional team-sport athletes and support staff.

### Virtual Training, Player Monitoring, and Continued Education

The current regulations enforced by national and international authorities limit professional team-sport athletes and support staff to travel and physically engage in standard training and recovery practices. However, the rapid emergence of sport technology in recent years (e.g., artificial intelligence, computer vision technology) may provide practical solutions to support athlete engagement and adherence in remote training interventions as well as telemedicine in the support of the overall COVID-19 risk screening process during and following the return to competition period. For instance, professional team-sport athletes and support staff may consider the use of low-cost, non-invasive, mobile-friendly, and publicly available software applications for the following purposes:

- Workout challenges and competitions (e.g., esports);
- Real-time display and exchange (e.g., leaderboards) of individual training results and progress between teammates, the community, as well as national and international governing entities (e.g., NBA);
- Remote training and recovery program design;
- Wellness and fatigue monitoring (e.g., customized forms, API integrations);
- Real-time video feedback on exercise technique and/or technical-tactical competencies;
- Training, recovery and lifestyle diaries, cognitive and physiological tests, and practical guides (Muaremi et al., 2013; Weber et al., 2019);
- Self-measurement of vertical jump performance (CMJ) as part of athlete monitoring, talent identification, and/or performance enhancement strategies (Balsalobre-Fernández et al., 2015);
- Self-measurement of power, force, velocity properties, and/or mechanical effectiveness in sprint running under field conditions as part of injury risk management as well as sprint training strategies (Romero-Franco et al., 2017);
- Physical contact tracing and competency management of risk mitigation procedures (Carmody et al., 2020) including mathematical models that incorporates the interaction of players during training sessions, leading to intra-club spreading, and during matches, responsible for inter-club contagions (Buld et al., 2020);
- Monitoring of respiratory frequency at rest and during exercise (Nicolò et al., 2017; Laveneziana et al., 2019) to analyze breathing patterns and physical effort (e.g., ergometry, pulsometry, sphygmomanometry, spirography).

Finally, continued growth and development opportunities for professional team-sport coaches and support staff may evolve around webinar software offering online education and communication tools while practicing social distancing. For instance, the “Coaches vs. COVID-19 webinar” endorsed by the NSCA is a free 4-day event in April 2020 that promotes the exchange of scientific ideas in strength and conditioning of athletes specifically pertaining to our current situation, and in turn, raise donations for those in the service industry and their families who have been impacted by COVID-19 (Coaches vs COVID-19., 2020).

## Conclusions

The COVID-19 outbreak has caused a chain reaction of unpredictable situations which challenge current professional team-sport athletes and support staff to remain emotionally stable and physically ready for the next steps in their career. However, it is the responsibility of all team-sport practitioners to practice good judgment in designing a safe and effective “return-to-competition action plan” for all of their athletes during these unprecedent times. Given that physical contact, social interaction, frequent air travel, and vigorous training sessions are likely unavoidable upon return to competition, the probability of immunodeficiency, and irreversibly, contraction and dissemination of contagious illnesses among athletes, support staff, family, and/or outreach communities, is sensible and realistic. For these reasons, professional team-sport organizations should be entitled to specifically tailored protection rules and regulations during and following the COVID-19 era. Particularly during the most intensive upcoming periods (e.g., preseason, training camps, back-to-back games, road trips), vigilance remains necessary from both an illness risk and injury risk perspective.

In essence, cross-training, sprint training, and high-intensity interval (HIIT) training sessions every second week of the “at-home period” may aid in the preservation of fundamental fitness qualities. From a loading perspective, the “50/30/20/10” and “FIT” rules may serve as a an appropriate guideline during and following return to competition. Additionally, injury and illness risk profiling should be encouraged to ensure maximal caution in each member of the organization, encompassing the combination of baseline and follow-up medical, sanitary, and fitness examinations. In addition, a personal “immunity scorecard” for each athlete and support staff member may help mitigate future risk of contraction and dissemination of the COVID-19 virus. This scorecard may present key indicators of immune system health (e.g., SIgA), prominent COVID-19 symptoms (e.g., core body temperature), inherited COVID-19 risk (e.g., age), as well as behavioral lifestyle parameters (e.g., sleep duration) and logistical constraints (e.g., travel duration). The assemblance and real-time visualization of this information would provide each athlete and staff member with meaningful and holistic insights, and encourages maximal caution, urgency, and compliance to preset rules across the entire organization. Finally, this brief opinion article provides practical suggestions based on five themes: periodization, exploration, virtual (at-home) training, player monitoring, and continued education. It is the aim of the authors to encourage researchers and practitioners to further expand upon these topics, ideas, and beliefs to successfully prepare professional team-sport athletes and support staff during and following the COVID-19 crisis. In the future, long-term rigorous surveillance of clinical outcomes will be needed in order to examine the precise impact these interventions have on professional team-sport athletes' health and performance, and in turn, finetune the ecological validity of current practices. Ultimately, this will help to ensure the safe global resurrection of a thriving professional team-sport community.

## Data Availability Statement

The original contributions presented in the study are included in the article/supplementary material, further inquiries can be directed to the corresponding author/s.

## Author Contributions

TH, SB, and JC-G: conceptualization and resources. SB and JC-G: methodology. TH: writing—original draft preparation. TH, SB, JC-G, and PA: review and editing. All authors have read and agreed to the published version of the manuscript.

## Conflict of Interest

The authors declare that the research was conducted in the absence of any commercial or financial relationships that could be construed as a potential conflict of interest.
